# Quinoa and Colonic Health: A Review of Bioactive Components and Mechanistic Insights

**DOI:** 10.3390/cimb47100815

**Published:** 2025-10-02

**Authors:** Yan Pan, Jimin Zheng, Zhixuan Wang, Shaohua Lin, Hongliang Jia, Hairun Pei, Ronghui Ju

**Affiliations:** 1 Beijing Vocational College of Agriculture, Beijing 102442, China; 2 Beijing Advanced Innovation Center for Food Nutrition and Human Health, Beijing Technology & Business University, Beijing 100048, China; 3 College of Chemistry, Beijing Normal University, Beijing 100875, China

**Keywords:** quinoa, colonic health, polysaccharides, gut microbiota modulation, colorectal cancer prevention

## Abstract

Quinoa (*Chenopodium quinoa* Willd.) is an ancient Andean crop renowned for its exceptional nutritional profile and diverse bioactive compounds, including polysaccharides, polyphenols, saponins, and essential fatty acids. As global incidence of colonic diseases such as inflammatory bowel disease (IBD), colorectal cancer (CRC), and celiac disease continues to rise, the therapeutic potential of quinoa has garnered increasing scientific attention. This review systematically examines the role of quinoa, with focus on quinoa polysaccharides (QPs), in maintaining and improving colonic health. It summarizes the molecular structure, functional properties, and gut microbiota-modulating effects of QPs, alongside emerging findings on their anti-inflammatory, antioxidant, immunomodulatory, and anticancer activities. Furthermore, the review explores quinoa’s auxiliary effects in mitigating CRC progression and chemotherapy resistance, alleviating intestinal inflammation, and supporting gastrointestinal integrity in celiac patients. By integrating evidence from multi-omics technologies, cell and animal models, and limited clinical studies with mechanistic insights, this review provides a focused synthesis of quinoa bioactive compounds in relation to colonic health. It highlights how precision nutrition and multi-omics approaches could guide future applications of quinoa as a novel functional food-based intervention for colonic diseases.

## 1. Introduction

### 1.1. The Importance of Colonic Health

As the terminal organ of the digestive system, the colon plays a crucial role not only in the absorption of nutrients but also as a key site of interaction between the gut microbiota and the host immune system. In recent years, the global incidence of colonic diseases has risen significantly. Among these, Inflammatory Bowel Disease (IBD) and Colorectal Cancer (CRC) have emerged as major public health concerns threatening human health. According to statistics, more than 3.83 million people worldwide are affected by IBD in 2021 [1], and there are approximately 1.9 million new CRC cases annually [2,3,4]. These diseases not only lower patients’ quality of life but also impose a substantial burden on healthcare systems.

Due to its safety and sustainability, dietary intervention has become an increasingly important strategy for managing colon health. Studies have shown that active components such as dietary fiber, polyphenols, and polysaccharides can improve colon health by modulating gut microbiota balance, enhancing intestinal barrier function, and inhibiting inflammatory responses [5,6]. Therefore, exploring the mechanisms by which novel functional food components influence colon-related diseases through gut microbiota holds significant practical value.

### 1.2. Introduction of Quinoa

Quinoa (Chenopodium quinoa Willd.), a member of the Chenopodiaceae family along with spinach and beet, is recognized as a “super grain” due to its high protein content and well-balanced amino acid profile [7,8,9]. In recent years, it has been promoted globally by the Food and Agriculture Organization (FAO) as a functional crop. Quinoa cultivation has expanded far beyond its native regions and is now grown in numerous countries across Europe, including France, England, Sweden, Denmark, the Netherlands, and Italy—as well as in China, India, Pakistan, New Zealand, Australia, Canada, the United States, and many others [10,11,12,13]. The Chenopodiaceae family, to which quinoa belongs, includes approximately 250 species worldwide [7,8,9,14]. Archeological evidence indicates that quinoa has been cultivated since as early as 5000 BC [8]. Its remarkable adaptability to diverse environments allows quinoa to thrive in a wide range of climates and geographic regions [11,15,16].

Quinoa seeds are widely recognized as an exceptionally nutritious grain, thanks to their relatively high protein content and superior protein quality, particularly in terms of essential amino acid composition, when compared to traditional cereals. Quinoa also contains essential fatty acids and a wide range of minerals [9,15]. It surpasses most cereals in levels of lipids, proteins, dietary fiber, and vitamins B1, B2, B6, C, and E, as well as minerals such as calcium, phosphorus, iron, and zinc. In addition, unlike many other grains, quinoa is naturally gluten-free and has demonstrated tolerability in celiac patients. This unique property underscores its value as a functional food for maintaining intestinal homeostasis and supports its potential for developing diverse, nutrient-rich products suitable for individuals with celiac disease [14].

Quinoa exists in both sweet and bitter varieties, classified according to their saponin content; varieties with less than 0.11% saponins are considered sweet [9,17]. Bioactive compounds in quinoa, such as flavonoids, phenolic acids, and saponins, are known to exhibit antimicrobial, antioxidant, and anti-inflammatory properties. In addition, quinoa seeds are rich in minerals, vitamins, and high-quality proteins, making quinoa a valuable ingredient in the development of functional foods aimed at supporting health and preventing disease [9,18].

With the rising global demand for functional foods, which may include conventional foods with specific health benefits or those enriched through natural processes, fortification, or technological modification [19,20], quinoa has garnered significant international interest for its nutritional profile, functional properties, and potential pharmaceutical applications [9,21,22,23].

Research on quinoa’s bioactive components has primarily focused on polysaccharides, saponins, polyphenols, and proteins. Among these, quinoa polysaccharides (QPs) have emerged as a key area of interest due to their structural diversity and wide range of biological activities. QPs have been shown to possess significant immunomodulatory [21,22], antioxidant [23], anticancer [24], and prebiotic properties [25,26]. For example, studies have demonstrated in vitro that QPs can enhance immune responses by activating macrophages, as well as alleviate oxidative stress-induced damage in intestinal epithelial cells [27]. What sets QPs apart from other plant-derived polysaccharides is their complex structural diversity, which includes α- and β-configured glucans and arabinoxylans [28]. These structures may exert specific regulatory effects on gut microbiota composition, such as promoting the growth of Bifidobacterium and Lactobacillus, thus contributing to their health benefits. However, research into the structure–function relationships and precise molecular targets of QPs remains in its early stages.

**Figure 1:**
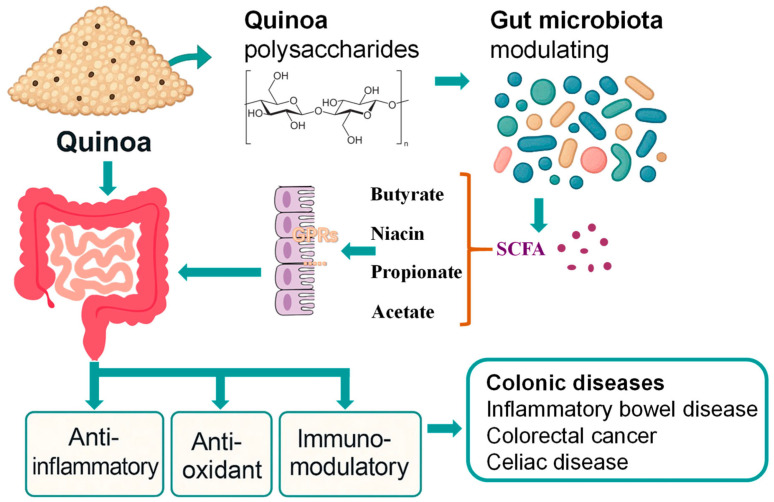
Quinoa’s mechanism of action on colonic health.

Compared with other functional grains such as barley or oats, quinoa contains a wide range of nutritional components, including saponins, proteins, carbohydrates, triterpenes, crude fiber, fatty acids, polysaccharides, and vitamins. Among these, several are closely linked to gut health and the prevention of intestinal diseases. Due to the growing attention surrounding quinoa polysaccharides, this review will first explore their impact on colon health. Subsequently, the effects of other bioactive compounds in quinoa will be discussed in relation to different types of colonic diseases (Figure 1).

## 2. Effects of Quinoa Polysaccharides on Colon Health

### 2.1. Structural and Functional Analysis of Quinoa Polysaccharides

**Figure 2:**
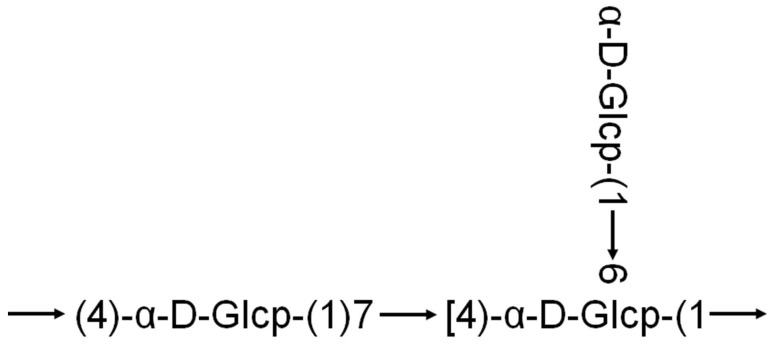
One possible structure of quinoa polysaccharide sugar residue linkages [30].

The biological activity of macromolecules is often closely related to their structural characteristics [29]. A study published in 2021 linked the antioxidant activity of quinoa polysaccharides to their molecular structure. Teng et al. [30] extracted two polysaccharide fractions (SQAP-1 and SQAP-2) from quinoa using alkaline extraction methods. Using nuclear magnetic resonance (NMR) and mass spectrometry (MS), they analyzed the structure of SQAP-2, which exhibited notable antioxidant activity. The backbone of SQAP-2 was suggested to be composed of Glc-(1→, →4)-Glcp-(1→, →4, 6)-Glcp-(1→ (Figure 2). Another study reported that quinoa polysaccharides composed of glucose, galactose, and arabinose formed triple-helix structures. These polysaccharides were shown to enhance the phagocytic activity of RAW264.7 macrophages and promote the secretion of TNF-α and IL-6 indicating a beneficial immune-stimulating effect activity [31]. In addition, research has demonstrated that the antioxidant capacity, anti-glycation activity, prebiotic potential, and immunostimulatory effects of pectic polysaccharides from quinoa are negatively correlated with their degree of esterification. Both native and modified pectic polysaccharides were found to exert immunostimulatory effects by activating the TLR4/NF-κB signaling pathway [27]. These findings suggest that quinoa polysaccharides may possess pectin-like adhesive properties, which could help form a protective gel layer in the gut (Table 1).

Overall, these studies contribute to a clearer understanding of the structure–function relationships of quinoa polysaccharides and support their potential application as functional food ingredients or nutritional enhancers.

### 2.2. Quinoa Polysaccharides and the Gut Microbiota

Polysaccharides are major macromolecules found in animals, plants, and the cell walls of microorganisms. They can be broadly classified into plant, animal, and microbial polysaccharides [11,33]. Recently, these compounds have drawn increasing scientific attention due to their diverse biological activities, including immunomodulatory, antioxidant, antiviral, anti-inflammatory, hypoglycemic, and prebiotic effects [11,34]. With advances in industrial applications, polysaccharides have become widely utilized across the food, pharmaceutical, and materials sectors [11]. Polysaccharides from natural sources have gained prominence for their ability to scavenge free radicals, inhibit lipid oxidation, enhance natural killer (NK) cell cytotoxicity, and activate macrophages and interleukins. These biological effects are closely linked to structural features such as monosaccharide composition, molecular weight, and glycosidic bond types [22,35,36,37].

As a primary energy source for gut microbes, the structure of polysaccharides plays a crucial role in shaping the gut microbiota. In recent years, there has been an explosion of research investigating the structure-function relationships between polysaccharides and microbial modulation. Notably, specific polysaccharide structures such as Rhamnogalacturonan-II (RG-II)—a highly branched pectic polysaccharide with 13 different sugars and 21 distinct glycosidic bonds—have been shown to be preferentially metabolized by *Bacteroides* species [38].

Another key mechanism involves the production of short-chain fatty acids (SCFAs), such as butyrate, acetate, and propionate, which are metabolic by-products of microbial fermentation of polysaccharides. SCFAs act both as energy substrates and signaling molecules. Butyrate, for instance, notably influence gene expression through histone deacetylase (HDAC) inhibition [39]. Several studies have shown that plant-derived polysaccharides, such as those from *Lycium barbarum* [40] and Astragalus [32], can modulate gut microbiota, enhance SCFA production, and improve intestinal barrier function. Importantly, quinoa polysaccharides also showed similar relationships, influencing their antioxidant, immunomodulatory and prebiotic activities.

The concept of the “microbiota–host metabolic axis,” introduced in 2021, highlights the role of specific microbes in producing functional metabolites—such as SCFAs, tryptophan derivatives, N-nitrosoproline, and HMPA—through dietary polysaccharide metabolism. These metabolites modulate host immunity and metabolic homeostasis [4,41,42]. For instance, *Bacteroides* can ferment polysaccharides into acetate [43,44], which binds to GPR43 receptors, inducing GLP-1 secretion, enhancing metabolic function, and reducing inflammation [45]. Butyrate production is also the result of cross-feeding between *Bifidobacterium* and butyrate-producing colonic bacteria, with the former producing lactate or acetate that fuels the latter. SCFAs activate GPR41/43 receptors on enteroendocrine and immune cells, inducing secretion of GLP-1/PYY and anti-inflammatory cytokines [41]. GLP-1 and GPR signaling improve glucose/lipid metabolism and gut homeostasis, while butyrate fuels colonocytes and upregulates genes for tight junctions and mucin production [45,46,47]. This cooperation supports gut barrier repair and illustrates a direct link between microbial genera and epithelial function [48]. Notably, the effects of SCFAs are dose-dependent and subject to interindividual variability, influenced by host genetics, microbiota composition, and dietary background, as discussed in Section 2.3.

Technological advances have also propelled gut microbiota research. One breakthrough is spatial transcriptomics, which revealed significant differences in cell types across gut regions and complex epithelial subtypes. It also identified how identical cell types organize into distinct neighborhoods, emphasizing the presence of specialized immune niches and linking genetic predispositions to gut diseases with specific cell types [49]. Another frontier is synthetic microbiota modeling. A Stanford research team constructed a well-defined synthetic microbial community—the most complex to date—and successfully transplanted it into mice, mimicking natural gut function. This milestone opens new avenues for engineering therapeutic microbial consortia [50]. Among these engineered microbes, *Akkermansia muciniphila* stands out. This mucin-degrading symbiont plays a vital role in maintaining intestinal homeostasis by modulating the mucus layer. With quantifiable health benefits and unique metabolic traits, *A. muciniphila* is now considered a model organism in microbiome research and shows great promise for disease therapy [51,52]. Its genome-editability further enables mechanistic studies of host–microbiota interactions, and probiotic formulations based on this species have shown clinical success in treating metabolic syndrome [53]. Another key innovation is the co-culture of gut organoids and bacteria. Nanjing Agricultural University recently developed a multilayered gut organoid–microbiota co-culture model, offering a novel platform to study host-microbe interactions at high resolution [54].

### 2.3. Challenges in Development of Quinoa Polysaccharides

From these advances, it becomes clear that gut dysbiosis is a multifaceted process involving complex regulatory mechanisms. The observable shifts in microbiota composition caused by quinoa polysaccharides support their functional relevance. However, major challenges remain in the study of quinoa polysaccharides and gut microbiota:

Lack of causal evidence: Most current studies rely on correlation analysis, with limited direct evidence (e.g., gnotobiotic or fecal microbiota transplantation models) to confirm the functional role of specific microbes.

High individual variability: Diet, genetics, and environmental factors introduce heterogeneity in microbiota responses, complicating precise intervention strategies.

Limited structure-function resolution: Many studies focus on crude polysaccharide extracts without isolating or characterizing specific active fractions (e.g., glycosidic linkages, branching patterns). While techniques like cryo-electron microscopy (cryo-EM) and single-cell metabolomics have advanced structural biology, their direct application to polysaccharides is limited due to the inherent heterogeneity and lack of defined tertiary structure. Instead, polysaccharide characterization typically employs methods such as 1D/2D NMR spectroscopy, GC-MS, MALDI-TOF-MS, and FTIR, which provide detailed insights into monosaccharide composition, linkage types, molecular weight distribution, and conformational features like triple-helix structures. However, structural studies of polysaccharides still rely on the innovation of new technologies.

Future research will likely move toward multi-omics integration, combining spatial transcriptomics and mass spectrometry imaging to reveal how quinoa polysaccharides affect the spatial distribution and function of gut metabolites in different colonic niches (e.g., crypts, lymphoid follicles). Single-cell technologies will also be employed to dissect how individual host cells respond to microbial metabolites. Additionally, deep learning models may be used to predict how specific polysaccharide structures influence microbial composition, accelerating the discovery of functional components.

## 3. The Auxiliary Role of Quinoa in Alleviating Intestinal Inflammation

Inflammation is clinically recognized as a physiological and pathological process characterized by redness, edema, fever, pain, and loss of function. Excessive or chronic inflammation, often accompanied by oxidative stress, plays a central role in the progression of various diseases, including autoimmune, neurological, cardiovascular disorders, and cancer [55,56,57]. While current treatments rely on steroidal and non-steroidal anti-inflammatory drugs (NSAIDs), their prolonged use is often associated with significant side effects [57,58]. This has prompted growing interest in natural compounds that exhibit antioxidant and anti-inflammatory properties, either individually or synergistically [57,59].

**Figure 3:**
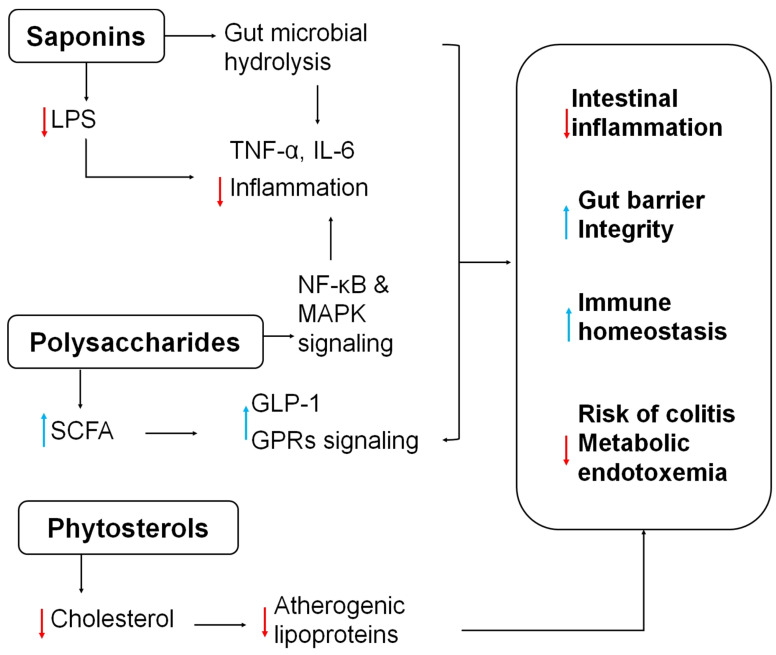
Mechanisms of Quinoa bioactive in Alleviating Intestinal Inflammation.

Quinoa, a pseudocereal traditionally used by the Andean people, has long been valued for its therapeutic effects. For example, black quinoa grains mixed with alcohol have been used in poultice form to treat muscle sprains, strains, and joint injuries, demonstrating its traditional anti-inflammatory applications [57]. Additionally, saponins present in quinoa seeds have been shown to inhibit the overproduction of inflammatory mediators such as nitric oxide, tumor necrosis factor (TNF), and IL-6, indicating their potential as bioactive compounds in functional foods aimed at inflammation control [28,60]. Quinoa saponins are largely unabsorbed in the small intestine and are hydrolyzed by gut microbes to sapogenins (and further to metabolites like ursodeoxycholic acid, UDCA) [61]. These microbial metabolites then reshape the microbiota (Figure 3) by enriching Lactobacillus and other butyrate-producing (anti-inflammatory) genera while suppressing Gram-negative LPS-producers [62]. This results in lower colonic LPS levels in enterocytes and immune cells, reducing pro-inflammatory cytokines (TNF-α, IL-6) [26,61]. Another study revealed that quinoa saponin ameliorated gut microbiota dysbiosis in DSS-treated mice. Metabolomic analysis identified UDCA as a key protective metabolite, which was subsequently shown to target the TLR4/NF-κB signaling pathway, enhancing colonic antioxidant activity and improving barrier integrity [63]. Quinoa saponins also modulate host metabolism of vitamin B_6_ and tryptophan in the gut, indirectly affecting immune homeostasis. Long-term low-dose saponin intake has been shown in rodents to improve insulin sensitivity and decrease adiposity via microbiota-mediated SCFA production and suppressed IL-6/LPS levels [26]. Although rodent studies offer important mechanistic insights, their relevance to human colonic health is limited by species-specific differences in metabolic pathways, gut microbiota profiles, and immune system function. Additional clinical investigations are required to assess whether similar effects occur in humans under comparable exposure conditions.

Phytosterols in quinoa have received relatively little attention despite their abundance. A 100 g portion of quinoa seeds can contain up to 118 mg of phytosterols, primarily β-sitosterol, campesterol, brassicasterol, and stigmasterol [14]. Ryan et al. [64] reported higher concentrations of β-sitosterol (63.7 mg/100 g), campesterol (15.6 mg/100 g), and stigmasterol (3.2 mg/100 g) in quinoa than in common grains such as barley, rye, millet, and corn. Phytosterols are lipophilic molecules structurally similar to cholesterol. Numerous epidemiological studies, intervention trials, and meta-analyses have confirmed their cholesterol-lowering capabilities [65,66]. Their mechanism (Figure 3) of action involves competing with cholesterol for absorption in the intestine and potentially suppressing the production of atherogenic lipoproteins in the liver and gut [67]. Furthermore, phytosterols have demonstrated anti-inflammatory, antioxidant, and anticancer properties [64].

Emerging research has also focused on quinoa-derived polysaccharides. In vitro studies using RAW264.7 macrophage cells have shown that non-starch quinoa polysaccharides (with proteins removed) stimulate cell proliferation via NF-κB and MAPK signaling pathways. These polysaccharides also promote CD40 expression and increase the production of nitric oxide and key cytokines, including IL-1β, IL-6, IL-10, and TNF-α, indicating notable immunomodulatory effects [68]. In an in vitro gut microbiota fermentation model, quinoa polysaccharides were found to selectively promote the growth of beneficial bacteria such as *Bifidobacterium* and *Actinobacteria*, while suppressing pathogenic *Escherichia coli* growth [69]. Animal studies further revealed that quinoa polysaccharides improve gut microbiota composition in rats fed a high-fat diet (HFD), reducing serum lipid levels and modulating key microbial phyla: decreasing Firmicutes and Bacteroidetes ratios, lowering Proteobacteria abundance, and restoring microbial balance. Notably, the reduction in the abundance of pro-inflammatory genera such as *Desulfovibrio* and *Enterobacter* correlated with decreased systemic inflammation [70]. In a dextran sulfate sodium (DSS)-induced mice colitis model, quinoa polysaccharide treatment alleviated disease symptoms, as indicated by a reduction in colon shortening and an increase in short-chain fatty acid (SCFA) production. These changes contributed to restoring gut microbiota balance and maintaining intestinal barrier integrity [71]. Overall (Figure 3), quinoa polysaccharides support gut health by shifting flora toward SCFA-producers, lowering pH and LPS, and participating SCFA–GPR pathways (mentioned in Section 2.2) to enhance immunity and epithelial resilience.

## 4. Quinoa’s Supportive Role in the Management of Celiac Disease

Quinoa’s exceptional nutritional value, medicinal properties, and natural gluten-free status make it a promising dietary component for a wide range of at-risk populations. These include children, the elderly, high-performance athletes, individuals with lactose intolerance, women susceptible to osteoporosis, and those with anemia, diabetes, dyslipidemia, obesity, or celiac disease [9,19,72]. In a clinical study involving 19 patients diagnosed with celiac disease, participants consumed 50 g of quinoa daily for six weeks as part of their gluten-free diet. Researchers evaluated gastrointestinal health markers, such as villus height-to-crypt depth ratio, surface enterocyte height, and intraepithelial lymphocyte count, as well as blood lipid profiles, both before and after the intervention [19,73,74]. The results showed that quinoa consumption improved gastrointestinal parameters, particularly restoring the villus height-to-crypt depth ratio from a suboptimal 2.8:1 to the normal 3:1. Importantly, the quinoa-enriched diet was well tolerated and did not exacerbate the disease. But the clinical study involved only 19 participants, and this small sample size limits the extent to which the findings can be extrapolated to broader populations. This constraint should be considered when clarifying the potential clinical relevance.

Additionally, serum lipid levels, including total cholesterol, LDL, HDL, and triglycerides, remained within healthy ranges, with slight decreases noted. These findings suggest a mild hypocholesterolemic effect, supporting quinoa’s role as a beneficial adjunct to the gluten-free diet in celiac patients. Overall, the addition of quinoa not only preserved intestinal integrity and immune balance but also contributed to improved histological and serological profiles.

The safety of quinoa varieties was evaluated for celiac patients by defining their protein profiles and their in vitro immune reactivity versus both anti-gliadin antibodies and IgAs from the celiac patient’s sera. Their findings suggest that 9 of the 12 varieties studies are safe for celiac individuals since a low binding-affinity of serum IgAs from celiac patients to proteins of these varieties was observed. The varieties PC1 (Lampa Grande), PC2 (Puno), and PC30 (commercial sample) exhibited mild positive immunoreactivity in the molecular weight area of gliadin. Therefore, these three varieties are considered less suitable for individuals with celiac disease. It is evident that the opportunity to include suitable quinoa in the formulation of gluten-free products will be inherently beneficial to celiac patients [73].

## 5. Quinoa’s Supportive Role in Colorectal Cancer Treatment

### 5.1. Overview of Colorectal Cancer

Cancer is a complex, multistage process involving a variety of genetic and epigenetic alterations. It often progresses silently, without symptoms, making early detection difficult [57,75,76,77]. Among various factors, diet plays a critical role in the development of cancer and other chronic diseases. Dietary phytochemicals, bioactive compounds found in plants, have shown strong cancer-preventive effects in both preclinical animal models and human epidemiological studies [57,77,78].

Colorectal cancer (CRC) ranks as the fourth most common cancer worldwide, causing approximately 550,000 deaths annually [79]. Its pathogenesis is multifactorial, involving genetic mutations and environmental influences, with poor dietary habits considered a major risk factor. Current standard treatments include surgery, chemotherapy, and radiation [80]. However, rising mortality rates suggest these approaches remain inadequate due to issues such as radiotherapy complications, toxicity, and severe side effects [81]. Alarmingly, recent epidemiological data indicate that CRC incidence is rising among younger populations, largely due to unhealthy dietary patterns [82,83]. This highlights the urgent need for novel therapeutic targets and supportive strategies.

### 5.2. The Role of Quinoa Fatty Acids in CRC Treatment

Quinoa contains 2.0–9.5% oil and is particularly rich in essential fatty acids such as linoleic and alpha-linolenic acids. It also boasts high levels of antioxidants like alpha-tocopherol, a form of vitamin E [8,14,84]. Saturated fatty acids such as palmitic acid make up about 10% of its total lipid content. The remaining 87.2–87.8% consists of unsaturated fatty acids, primarily oleic acid (19.7–29.5%), linoleic acid (49.0–56.4%), and alpha-linolenic acid (8.7–11.7%)—a profile comparable to soybeans [8,16]. The total lipid content of quinoa is approximately 14.5%, with about 70% being unsaturated fatty acids. These are well-preserved by vitamin E, which serves as a natural antioxidant [9,85].

**Figure 4:**
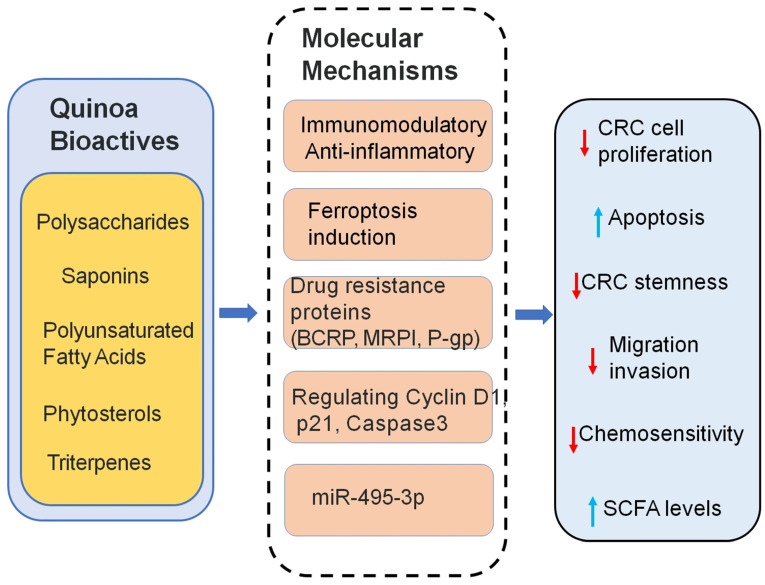
Mechanistic pathways of Quinoa bioactive in CRC prevention and therapy.

Polyunsaturated fatty acids (PUFAs), which cannot be synthesized by the human body, are essential for reducing blood lipids, modulating inflammation, and preventing cancer. Quinoa is a rich source of such PUFAs. Recent studies investigated quinoa-derived PUFAs (QPA) and their ability to counteract drug resistance in CRC [86]. QPAs were found to suppress the expression of drug resistance proteins (P-gp, MRP1, and BCRP), thereby increasing the sensitivity of resistant CRC cells to chemotherapy. Additionally, QPAs inhibited cancer stemness by downregulating the stem cell marker CD44 and induced ferroptosis, a form of iron-dependent cell death, by suppressing SLC7A11 expression, reducing ferritin levels, and increasing intracellular iron concentrations. These dual actions, suppressing stemness and inducing ferroptosis, suggest that QPAs may serve as potential chemosensitizers. Gas chromatography analysis identified key fatty acid components in quinoa responsible for these effects. Unlike many plant-derived compounds that trigger only one pathway, QPAs offer a unique dual mechanism (Figure 4). While these findings are promising, it is important to note that they are based on preclinical data. Further studies, including in vivo and clinical investigations, are needed to validate the therapeutic relevance. Nutritional supplementation with PUFAs during cancer treatment may not only provide health benefits but also enhance the efficacy of chemotherapy [87,88]. This study underscores the potential of quinoa PUFAs as functional food-based adjuncts in anticancer therapy.

### 5.3. Quinoa Saponins in CRC Therapy

Quinoa saponins have demonstrated the ability to inhibit the survival of certain cancer cell types. However, studies specifically examining their effects on CRC cells are limited. In vitro research confirmed that quinoa saponins significantly suppress the growth of human CRC HT-29 cells [89]. MTT assays showed that saponins reduced cell proliferation. After treatment with 40 μg/mL of saponins, the proportion of cells in the G0/G1 phase increased by 22.97%, and the apoptosis rate rose by 22.55% compared to controls. This cell cycle arrest was mediated by downregulating Cyclin D1 and upregulating p21. Apoptosis was further promoted through the activation of Caspase-3 and Bax, and suppression of Bcl-2. Autophagy was also induced, as shown by the increased expression of LC-3II and Beclin1, with autophagy inhibition significantly reducing the anti-proliferative effect (Figure 4). Moreover, saponins impaired HT-29 cell migration, reducing wound healing and migration ability by 38.21% and 69.48%, respectively. These effects were linked to increased E-cadherin and decreased N-cadherin expression. Importantly, all these changes were dose-dependent. Together, these findings provide initial support for the anticancer potential of quinoa saponins.

### 5.4. Quinoa Triterpenes in CRC Therapy

Triterpenes are a major group of anticancer phytochemicals found in many natural products. Quinoa contains a variety of terpenes, including monoterpenes, sesquiterpenes, and predominantly triterpenes. However, their anticancer effects, particularly against CRC, remain underexplored [90]. Recent studies have isolated a water-soluble triterpene, QBT, from quinoa bran using acetone extraction and macroporous resin separation (AB-8 column). Unlike most triterpenes, QBT is water-soluble, enhancing its bioavailability and clinical potential. In vitro and in vivo experiments demonstrated that QBT significantly inhibited CRC cell proliferation and promoted apoptosis. QBT also downregulated drug resistance proteins and enhanced the chemosensitivity of resistant CRC cells. Mechanistically (Figure 4), this was linked to its ability to reduce methylation of the miR-495-3p promoter region [91]. These findings highlight the therapeutic promise of QBT and underscore quinoa’s potential as a functional food source in integrative cancer treatment strategies.

### 5.5. Quinoa Polysaccharides in CRC Therapy

Quinoa polysaccharides (QPs) have shown promising potential as functional bioactive compounds in CRC therapy. Recent in vitro studies have demonstrated that purified QPs can inhibit the proliferation of cancer cells, including SMMC-7721 and MCF-7, in a dose- and time-dependent manner. Importantly, QPs exhibited minimal cytotoxicity toward normal cell lines (L02 and MCF-10A), indicating favorable biosafety profiles. Additionally, QPs exerted immunomodulatory and anti-inflammatory effects, as evidenced by its ability to promote RAW264.7 macrophage proliferation at higher concentrations (25–100 μg/mL) and inhibit LPS-induced nitric oxide (NO) production in these cells. These dual functions (Figure 4), immune enhancement and inflammation suppression, highlight the therapeutic potential of QPs not only as a direct anticancer agent but also as a supportive compound to enhance host immunity and reduce inflammation in the tumor microenvironment. Overall, quinoa-derived polysaccharides may offer a natural, safe, and multifunctional strategy for CRC prevention and adjunct treatment [24].

## 6. Conclusions

Quinoa emerges as a promising functional food with multifaceted benefits for colonic health, owing to its rich array of bioactive components (Table 2). Polysaccharides demonstrate significant potential in modulating gut microbiota, enhancing immune responses, suppressing inflammation, and exerting protective effects against colorectal cancer. Other constituents such as saponins, phytosterols, polyunsaturated fatty acids, and triterpenes further contribute to quinoa’s therapeutic profile by targeting pathways involved in tumor suppression, ferroptosis induction, and gut barrier repair. These pathways encompass the suppression of TLR4/NF-κB signaling, activation of antioxidant responses, and stimulation of SCFA-mediated GPR41/43 signaling cascades. Clinical studies also support quinoa’s safety and efficacy in celiac disease management, highlighting its role in restoring intestinal morphology and metabolic homeostasis. These multifaceted mechanisms support the therapeutic potential of quinoa in maintaining intestinal integrity, modulating immunity, and preventing inflammation-related pathologies. Despite these advances, challenges remain in deciphering the structure–function relationships of quinoa components and establishing causality in microbiota-mediated effects.

**Table 2:** Bioactive components in quinoa and their colonic health functions.

Component	Key Pathway	Biological Function	Target Outcome
Polysaccharides	SCFA-GPRs activation HDAC inhibition TLR4/NF-κB	Immunomodulation, gut microbiota modulation, SCFA production	Alleviate colitis Enhance gut integrity Improved barrier integrity
Saponins	LPS suppression Regulating Cyclin D1, p21, Caspase3	Anti-inflammatory, induce apoptosis and autophagy in CRC cells	Suppress CRC cell growth and migration
Polyunsaturated Fatty Acids (PUFAs)	Ferroptosis induction via SLC7A11 inhibition	Ferroptosis induction, downregulate drug-resistance protein	Reverse chemoresistance in CRC
Phytosterols	Cholesterol-lowering	Suppressing atherogenic lipoproteins	Anti-inflammatory cardiometabolic support
Triterpenes	miR-495-3p demethylation	miRNA modulation, apoptosis induction	Inhibit CRC progression
Natural gluten-free	Gluten-free Remain serum lipid levels	Intestinal homeostasis improves histological and serological profiles	Gluten-free diet in celiac patients

Quinoa’s direct application as a treatment in colonic health still faces certain challenges. Although many in vivo and preclinical studies involving quinoa have been conducted, only a limited number specifically address colonic health in animal or human clinical models (Table 3). Table 3 reveals a preclinical evidence base: animal models account for most studies that directly evaluate quinoa bioactivity compounds in colonic health, while human data remain scarce and largely limited to small tolerability treatment in celiac patients rather than therapeutic efficacy. These results demonstrate a translational gap between preclinical findings and human applications. Moreover, Table 3 illustrates that different compounds exert distinct but potentially complementary actions, suggesting that multi-compound synergies may play an important role. This reinforces the urgent need for rigorously designed human clinical research to confirm the translational value of quinoa bioactive compounds in colonic health.

Furthermore, the integration of precision nutrition and multi-omics approaches will be crucial for advancing quinoa research in colonic health. Precision nutrition frameworks can help identify which subgroups of patients are most likely to benefit from quinoa. Multi-omics strategies, including metabolomics, transcriptomics, and metagenomics, can provide systems-level insights into the mechanisms of action. For example, metabolomics has already revealed UDCA as a key metabolite mediating the anti-colitis effects of quinoa saponins, while transcriptomic profiling could clarify how quinoa polysaccharides modulate epithelial barrier and inflammatory signaling pathways. Metagenomic analyses may further delineate how quinoa dietary fibers reshape microbial networks toward SCFA-producing communities. Future research should emphasize precision nutrition approaches, multi-omics integration, and clinical validation to unlock the full potential of quinoa-based interventions in the prevention and treatment of colonic diseases.

## Figures and Tables

**Table 1:** Quinoa Polysaccharides: Structural Characteristics and Functional Implications.

Structural Feature	Functional Implication	Supporting Studies
Molecular weight (10–100 kDa)	Influences fermentation rate and SCFA production	[30,31,32]
Arabinose/Galactose branching	Enhances immunomodulatory activity	[27,28]
Triple-helix conformation	Improves antioxidant and prebiotic effects	[30,31]
Degree of esterification	Negatively correlated with anti-glycation and immunostimulation	[27]
α-/β-configured glycosidic linkages	Determines microbiota selectivity	[28]

**Table 3:** Clinical and Animal Studies on Quinoa Bioactive in Colonic Health.

Disease	Study Type	Quinoa Component	References
Celiac disease	Clinical	Whole quinoa (gluten-free diet)	[74]
Colitis/gut inflammation (DSS, LPS, systemic models)	Animal	Polysaccharides	[60,69,70]
		Saponins	[61,63]
		Bran soluble dietary fiber	[71]
Colorectal cancer	Animal	PUFAs	[86]
		Terpenoids	[90,91]

## Data Availability

The original contributions presented in this study are included in the article. Further inquiries can be directed to the corresponding author(s).

## Abbreviations

The following abbreviations are used in this manuscript:

IBD	Inflammatory bowel disease
CRC	Colorectal cancer
QPs	Quinoa polysaccharides
FAO	Food and Agriculture Organization
NMR	Nuclear magnetic resonance
MS	Mass spectrometry
NK	Natural killer
SCFAs	Short-chain fatty acids
GLP-1	Glucagon-like peptide-1
LPS	Lipopolysaccharide
HDAC	Histone deacetylase
NSAIDs	Non-steroidal anti-inflammatory drugs
DSS	Dextran sulfate sodium
PUFAs	Polyunsaturated fatty acids
P-gp	P-glycoprotein
MRP1	Multidrug Resistance-associated Protein 1
BCRP	Breast Cancer Resistance Protein
QBT	Quinoa-derived terpenoid compounds
